# Mutant Phenotype p53 Immunohistochemical Expression Is Associated With Poor Prognostic Parameters and Disease-Free Survival in Triple-Negative Metaplastic Breast Carcinoma

**DOI:** 10.7759/cureus.15244

**Published:** 2021-05-25

**Authors:** Atif A Hashmi, Alina Sajid, Muzna Hussain, Shamail Zia, Sabeeh Islam, Muhammad Asad Diwan, Syed Munqaad Ali, Muhammad Irfan, Farozaan Shamail, Fazail Zia

**Affiliations:** 1 Pathology, Liaquat National Hospital and Medical College, Karachi, PAK; 2 Internal Medicine, Sharif Medical and Dental College, Lahore, PAK; 3 Cardiovascular Medicine, Cleveland Clinic, Cleveland, USA; 4 Pathology, Ziauddin University, Karachi, PAK; 5 Internal Medicine, Faisalabad Medical University, Faisalabad, PAK; 6 Pathology, Aga Khan University, Karachi, PAK; 7 Internal Medicine, Dow University of Health Sciences, Karachi, PAK; 8 Statistics, Liaquat National Hospital and Medical College, Karachi, PAK; 9 Pathology, Jinnah Sindh Medical University, Karachi, PAK

**Keywords:** metaplastic breast carcinoma, triple-negative breast carcinoma, p53, mutant phenotype, breast cancer

## Abstract

Introduction

Metaplastic breast carcinoma (MBC) is a special type of breast cancer that is most commonly triple-negative and has the worst outcome compared to other breast tumors. p53 is a tumor suppressor gene that is frequently mutated in many human cancers. The association of mutant p53 immunohistochemical expression with clinical and prognostic parameters has not been widely studied in triple-negative MBC. Therefore, in this study, we evaluated the expression patterns of p53 in triple-negative MBC and its association with clinical and prognostic parameters.

Methods

A retrospective observational study was conducted in the Department of Histopathology at Liaquat National Hospital and Medical College, Pakistan, for a duration of 11 years. A total of 101 cases of triple-negative MBCs were included in the study. p53 immunohistochemistry was performed on the representative tissue blocks. Cases with diffuse strong positive p53 expression were labeled mutant phenotype, while cases with weak patchy p53 expression were termed wild-type.

Results

The mean age of the patients was 48.33±11.47 years, and the mean tumor size was 3.98±2.07 cm. The mean Ki67 index was 48.98±22.97%. The median disease-free survival of the patients was 24 (three to 68) months, with a median follow-up of 37 (13 to 77) months. Most of the cases were tumor (T)-stage II (51.5%). Axillary metastasis was present in 36.6% of cases, with the perinodal extension in 16.8% of cases. Most cases were non-basal subtype (91.1%), and the majority of cases were grade III (85.1%). Recurrence was observed in 17.8% of cases. Among 101 cases, 52.5% cases showed mutant phenotype p53 expression. A significant association of p53 expression was noted with tumor grade, Ki67 index and disease-free survival. Cases with mutant phenotype p53 expression had a higher tumor grade, higher Ki67 index, and poorer disease-free survival than cases with wild-type p53 expression.

Conclusion

A substantial proportion of triple-negative MBC expressed diffuse strong expression (mutant phenotype) of p53 in our study, signifying a potential role of p53 as a therapeutic target in triple-negative MBC. Moreover, association of p53 with poor prognostic parameters, such as higher tumor grade and Ki67, and poor disease-free survival underscores the prognostic significance of p53 in triple-negative MBC.

## Introduction

Triple-negative breast cancers (TNBCs) are defined by the lack of expression of estrogen receptor (ER), progesterone receptor (PR), and human epidermal growth factor receptor 2 (HER2/neu). Compared with luminal and HER2/neu positive breast cancers, they are considered to have a poor outcome [1,2]. Metaplastic breast carcinoma (MBC) is a special type of breast cancer that is most commonly triple-negative and thus has limited therapeutic options [3,4]. p53 is a tumor suppressor gene that is frequently mutated in many human cancers. A mutated p53 gene leads to production of a truncated protein that is indigestible inside the cancer cell, and therefore remains inside the cell for a longer period. Normal human cells show weak heterogeneous expression of p53 protein by immunohistochemistry (IHC), referred to as wild-type expression. Alternatively, a mutated p53 gene leads to abnormal production of p53 protein that gives a strong and diffuse p53 expression called mutant phenotype. Previous studies have confirmed that this diffuse strong p53 expression correlates with p53 gene mutation on a molecular level [5]. Association of mutant p53 immunohistochemical expression with clinical and prognostic parameters has not been widely studied in triple-negative MBC. Therefore, in this study, we evaluated the expression patterns of p53 in triple-negative MBC and its association with clinical and prognostic parameters.

## Materials and methods

A retrospective observational study was conducted in the Department of Histopathology at Liaquat National Hospital and Medical College, Pakistan, for a duration of 11 years. A total of 101 cases with histopathological diagnosis of MBC, negative ER, PR and HER2/neu IHC were included in the study. Cases that received neoadjuvant chemotherapy were excluded from the study, along with cases with positive ER, PR or HER2/neu expression. Hematoxylin and eosin (H&E)-stained slides of all cases were retrieved and diagnoses were reviewed. Moreover, ER, PR and HER2/neu immunohistochemical studies were performed to confirm the triple-negative status, as described in previous studies [6-10]. Cytokeratin 5/6 (CK5/6) was performed to subtype triple-negative MBCs into basal and non-basal subtypes. Cases with positive CK5/6 were termed as basal phenotype triple-negative MBC. Ki67 was performed to determine the proliferative index of the tumor. More than 1% nuclear expression of ER and PR in invasive cancer cells was taken as positive ER/PR expression. Moderate to strong membranous expression of HER2/neu in more than 10% invasive cancer cells was taken as positive Her2/neu expression. The average percentage of positively stained cancer cells (nuclear expression) was recorded to determine the Ki67 proliferative index. Moderate to strong cytoplasmic expression of CK5/6 was interpreted as positive expression.

**Figure 1:**
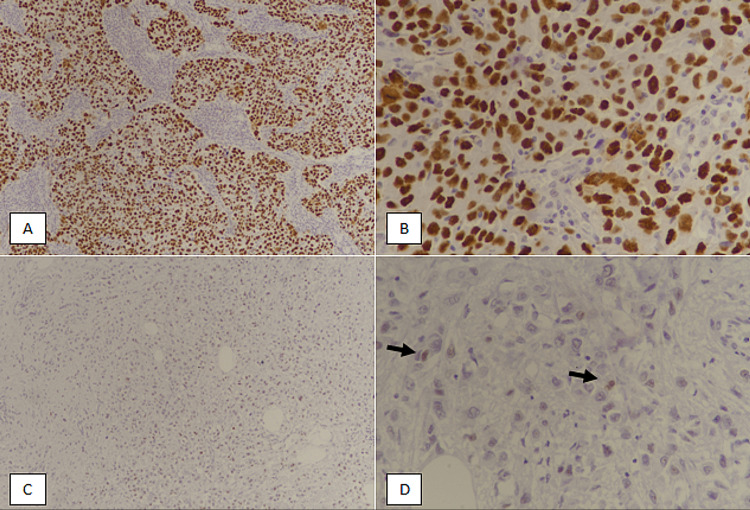
p53 expression in triple-negative metaplastic breast carcinoma. (A): IHC-staining at 100x magnification showing diffuse strong nuclear expression of p53 (mutant phenotype). (B): Mutant phenotype p53 expression at 400x magnification. (C): IHC-staining at 100x magnification depicting weak patchy p53 expression (wild-type). (D): Wild-type p53 expression at 400x magnification. Few tumor cells show weak nuclear staining (arrows). IHC: immunohistochemical

p53 IHC was performed on representative tissue blocks, as described in previous studies [11-13]. Cases with diffuse strong positive p53 expression were labeled mutant phenotype, while cases with weak patchy p53 expression were termed wild-type (Figure 1A, 1B).

Data analysis was performed using SPSS Statistics version 26.0 (IBM Inc., Armonk, NY, USA). Chi-square, independent t-test, and Fisher’s exact tests were used to check the association. Survival analysis was done by the Kaplan-Meier method. P-values < 0.05 were considered significant.

## Results

The mean age of the patients was 48.33±11.47 years and the mean tumor size was 3.98±2.07 cm. The mean Ki67 index was 48.98±22.97%. The median disease-free survival of the patients was 24 (three to 68 months), with a median follow-up of 37 (13 to 77) months. Most of the cases were tumor (T)-stage II (51.5%). Axillary metastasis was present in 36.6% of cases, with the perinodal extension in 16.8% of cases. In situ component was present in 37.6% of cases. Lymphovascular invasion was present in 22.8% of cases. Most cases were non-basal subtype (91.1%), and the majority of cases were grade III (85.1%). Recurrence was observed in 17.8% of cases. Among 101 cases, 52.5% cases showed mutant phenotype p53 expression (Table 1).

**Table 1:** Clinicopathological features of studied population SD, standard deviation; T, tumor; N, nodal

Clinicopathological parameters	Values
Age (years), mean±SD	48.33±11.47
Tumor size (cm), mean±SD	3.98±2.07
Ki67 index (%), mean±SD	48.98±22.97
Disease-free survival (months), median (range)	24 (3–68)
T-stage	
T1, n (%)	20 (19.8)
T2, n (%)	52 (51.5)
T3, n (%)	29 (28.7)
Axillary metastasis	
Present, n (%)	37 (36.6)
Absent, n (%)	64 (63.4)
N-stage	
N0, n (%)	64 (63.4)
N1, n (%)	20 (19.8)
N2, n (%)	6 (5.9)
N3, n (%)	11 (10.9)
Perinodal extension	
Present, n (%)	17 (16.8)
Absent, n (%)	84 (83.2)
Necrosis	
Absent, n (%)	15 (14.9)
Focal, n (%)	59 (58.4)
Extensive, n (%)	27 (26.7)
Fibrosis	
Mild, n (%)	26 (25.7)
Moderate, n (%)	57 (56.4)
Severe, n (%)	18 (17.8)
Lymphocytic infiltration	
Absent, n (%)	9 (8.9)
Focal, n (%)	78 (77.2)
Extensive, n (%)	14 (13.9)
Insitu component	
Present, n (%)	38 (37.6)
Absent, n (%)	63 (62.4)
Lymphovascular invasion	
Present, n (%)	23 (22.8)
Absent, n (%)	78 (77.2)
Dermal lymphatic invasion	
Present, n (%)	9 (8.9)
Absent, n (%)	92 (91.1)
Pagetoid spread	
Present, n (%)	2 (2)
Absent, n (%)	99 (98)
Triple-negative subtype	
Basal, n (%)	9 (8.9)
Non-basal, n (%)	92 (91.1)
Chemotherapy	
Yes, n (%)	98 (97)
No, n (%)	3 (3)
Radiation	
Yes, n (%)	69 (68.3)
No, n (%)	32 (31.7)
Tumor grade	
Grade II, n (%)	15 (14.9)
Grade III, n (%)	86 (85.1)
Survival status	
Alive, n (%)	82 (81.2)
Expired, n (%)	19 (18.8)
Recurrence	
Yes, n (%)	18 (17.8)
No, n (%)	83 (82.2)
p53	
Wild phenotype, n (%)	48 (47.5)
Mutant phenotype, n (%)	53 (52.5)

Table 2 shows the association of p53 expression with clinicopathological parameters. A significant association of p53 expression was noted with tumor grade, Ki67 index and disease-free survival. Cases with mutant p53 phenotype had a higher tumor grade, higher Ki67 index, and poorer disease-free survival than cases with wild-type p53 expression. Alternatively, no significant association of p53 expression was noted with age, tumor size, T-stage, N-stage and triple-negative subtype.

**Table 2:** Association of clinicopathological features with p53 expression *Independent t-test was applied, **Chi-square test was applied, ***Fisher’s exact test was applied, ****Significant as <0.05 SD, standard deviation; T, tumor; N, nodal

Clinicopathological parameters	Values		P-value
	p53 expression		
	Wild Phenotype	Mutant Phenotype	
Age (years), mean±SD*	49.25±11.22	47.49±11.74	0.444
Tumor size(cm), mean±SD*	4.33±2.22	3.66±1.89	0.109
Ki67 index (%), mean±SD*	44.17±22.41	53.34±22.80	0.044****
Disease-free survival (months), mean±SD*	33.15±15.08	20.45±6.93	<0.0001****
T-stage**			
T1, n (%)	7 (14.6)	13 (24.5)	0.139
T2, n (%)	23 (47.9)	29 (54.7)	
T3, n (%)	18 (37.5)	11 (20.8)	
Axillary metastasis**			
Present, n (%)	18 (37.5)	19 (35.8)	0.863
Absent, n (%)	30 (62.5)	34 (64.2)	
N-stage***			
N0, n (%)	30 (62.5)	34 (64.2)	0.337
N1, n (%)	12 (25)	8 (15.1)	
N2, n (%)	1 (2.1)	5 (9.4)	
N3, n (%)	5 (10.4)	6 (11.3)	
Perinodal extension**			
Present, n (%)	6 (12.5)	11 (20.8)	0.268
Absent, n (%)	42 (87.5)	42 (79.2)	
Triple-negative subtype***			
Basal, n (%)	3 (6.3)	6 (11.3)	0.493
Non-basal, n (%)	45 (93.8)	47 (88.7)	
Tumor grade**			
Grade II, n (%)	11 (22.9)	4 (7.5)	0.030****
Grade III, n (%)	37 (77.1)	49 (92.5)	
Survival status**			
Alive, n (%)	43 (89.6)	39 (73.6)	0.040****
Expired, n (%)	5 (10.4)	14 (26.4)	
Recurrence**			
Yes, n (%)	8 (16.7)	10 (18.9)	0.773
No, n (%)	40 (83.3)	43 (81.1)	

**Figure 2:**
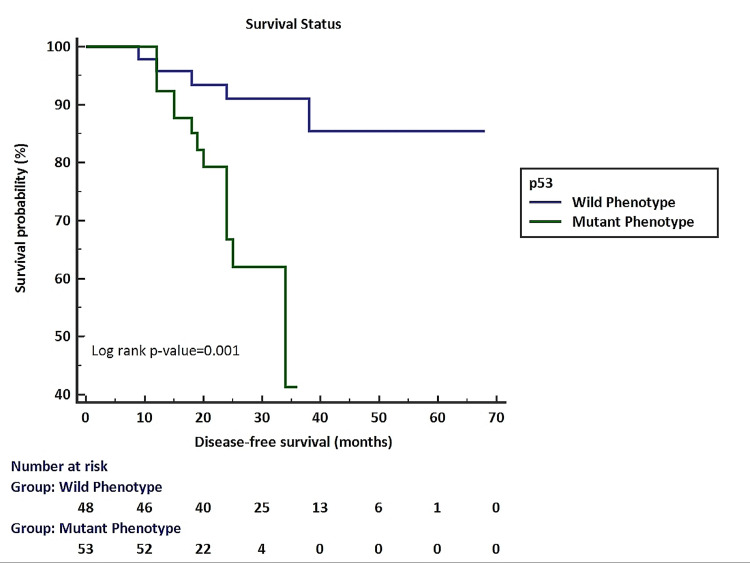
Association of p53 expression with disease-free survival by Kaplan–Meier method

Figure 2 shows the association of p53 expression with disease-free survival by Kaplan-Meier analysis. A significant association of p53 expression was noted with disease-free survival. Poor disease-free survival was noted in cases with mutant p53 phenotype.

## Discussion

In this study, we found that a significant percentage of cases of triple-negative MBC had mutant phenotype p53 expression. Moreover, mutant phenotype p53 expression was significantly associated with poor prognostic parameters such as higher tumor grade and higher mean Ki67 index. Furthermore, mutant phenotype p53 expression was associated with shorter disease-free survival, thus suggesting a prognostic value of p53 in triple-negative MBC.

MBCs are characterized by a carcinomatous component (CC) that can be in situ or invasive, along with a heterogeneous sarcomatous component (HSC). Lien et al. investigated p53 mutation patterns in both CC and HSC of MBCs and identified identical p53 mutations in both components, suggesting a monoclonal histogenesis of both components. Furthermore, based on the patterns of p53 mutations, they concluded that p53 mutation is an early event in MBC, before the invasion, and it is sustained throughout the tumor progression [14].

Li et al., in a study involving a cohort of 278 cases of TNBC, identified p53 immunoexpression in 58.6% of cases. Concordant with our findings, they also found a significant association of p53 expression with worse overall survival and poor disease-free survival [15]. Yang et al. also found that p53 positivity was predictive of outcome in breast cancer patients with visceral metastasis [16].

We found 52.5% positivity for p53 protein (mutant phenotype) in triple-negative MBC. Comparable frequencies of p53 expression were found in other studies [15,16]. Duffy et al. discussed the potential role of various compounds that can reactivate mutant p53 protein and convert it to acquire wild-type properties, thus signifying mutant p53 as a potential therapeutic target [17]. Similarly, Synnott et al. identified an anti-p53 drug targeting mutant p53 in TNBC [18].

Our study has a few limitations, such as not performing molecular studies to identify p53 mutations. Moreover, single-institution data further limit the value of the study. However, our findings signify a prognostic value for p53 expression in triple-negative MBC.

## Conclusions

We found p53 IHC to be of prognostic value in triple-negative MBC, owing to the significant association of mutant phenotype p53 expression with higher tumor grade and Ki67 index, and poorer disease-free survival. Furthermore, as a significant proportion of triple-negative MBC had mutant phenotype p53 expression, p53 may serve as a potential therapeutic target. However, large-scale prospective cohort studies are recommended to evaluate its significance as a therapeutic biomarker. Moreover, molecular correlation of immunohistochemical p53 expression is also needed with p53 gene mutations.
